# Adverse effects

**DOI:** 10.1186/1745-6215-12-S1-A9

**Published:** 2011-12-13

**Authors:** Stephen JW Evans

**Affiliations:** 1 Dept of Medical Statistics, London School of Hygiene & Tropical Medicine, London WC1E 7HT, UK

## 

Unwanted effects can occur with all medical interventions and most of these are adverse but this paper concentrates on adverse effects in randomised controlled trials (RCTs) of drug treatments. The process relies on a patient or health professional noticing and recording an adverse effect. Randomisation is the main tool for inferring causality and distinguishing between adverse events (co-incidental) and reactions (causal). The process requires careful recording as well as appropriate analysis and reporting of potential adverse drug reactions (ADRs).

Major statistical issues that arise are the recording process which should obtain unbiased data, and the problems of multiple possible ADRs. Multiplicity is a general problem in analysis of RCTs. Efficacy analyses have largely dealt with such problems by pre-specifying primary outcomes and secondary outcomes, though the use of composite outcomes and multiple “primary” outcomes, together with considerable numbers of secondary ones suggests that the issues are not resolved even for efficacy.

Pre-specification of adverse effects can rarely be done, at least not with any completeness. New ADRs that may be relatively rare may surprise investigators by their occurrence. Very much more statistical effort has been applied in analysis of efficacy than for analysis of harms, yet it is the absence of harm (safety) that is a primary concern of patients. Evidence both from surveys and even trials show that many patients have unrealistic expectations regarding benefits and the (total) absence of harms [1].

Conventional (Bonferroni) corrections for multiplicity may make the type II error for harms unacceptably high. Bayesian Hierarchical Models [2] have the potential to address these problems in RCTs as well as in observational studies and spontaneous reporting. Patients’ needs must be met by applying the most effective methods of analysis (e.g. Kaplan-Meier plots) [3], clear reporting [4] and sensible interpretation to the ADRs seen in trials.
